# A Source Anonymity-Based Lightweight Secure AODV Protocol for Fog-Based MANET

**DOI:** 10.3390/s17061421

**Published:** 2017-06-17

**Authors:** Weidong Fang, Wuxiong Zhang, Jinchao Xiao, Yang Yang, Wei Chen

**Affiliations:** 1 Key Laboratory of Wireless Sensor Network & Communication, Shanghai Institute of Micro-system and Information Technology, Chinese Academy of Sciences, Shanghai 201800, China; weidong.fang@mail.sim.ac.cn (W.F.); yang.yang@mail.sim.ac.cn (Y.Y.); 2 Shanghai Research Center for Wireless Communication, Shanghai 201210, China; 3 Guangzhou Shenyang Institute of Automation, Chinese Academy of Sciences, Guangzhou 511458, China; xiaojinchao@sia.cn; 4 School of Computer Science and Technology, China University of Mining and Technology, Xuzhou 221116, China; chenw@cumt.edu.cn

**Keywords:** mobile ad hoc network, fog computing, security, AODV, energy efficiency

## Abstract

Fog-based MANET (Mobile Ad hoc networks) is a novel paradigm of a mobile ad hoc network with the advantages of both mobility and fog computing. Meanwhile, as traditional routing protocol, ad hoc on-demand distance vector (AODV) routing protocol has been applied widely in fog-based MANET. Currently, how to improve the transmission performance and enhance security are the two major aspects in AODV’s research field. However, the researches on joint energy efficiency and security seem to be seldom considered. In this paper, we propose a source anonymity-based lightweight secure AODV (SAL-SAODV) routing protocol to meet the above requirements. In SAL-SAODV protocol, source anonymous and secure transmitting schemes are proposed and applied. The scheme involves the following three parts: the source anonymity algorithm is employed to achieve the source node, without being tracked and located; the improved secure scheme based on the polynomial of CRC-4 is applied to substitute the RSA digital signature of SAODV and guarantee the data integrity, in addition to reducing the computation and energy consumption; the random delayed transmitting scheme (RDTM) is implemented to separate the check code and transmitted data, and achieve tamper-proof results. The simulation results show that the comprehensive performance of the proposed SAL-SAODV is a trade-off of the transmission performance, energy efficiency, and security, and better than AODV and SAODV.

## 1. Introduction

**Figure 1:**
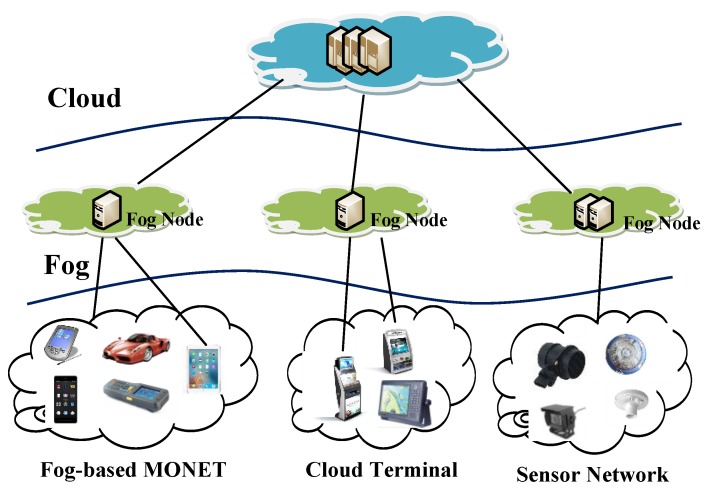
Architecture of the Fog-based MANET.

Every application and system that is managed in a cloud and mobile cloud service is promising for big data processing. However, centralized cloud computing is unnecessary and inefficient for the management and application in a large-scale mobile ad hoc network. To relieve the computation and communication burden on mobility management in cloud computing, fog computing can be adopted. Fog computing was firstly proposed by Cisco in 2012 [1]. It is an extension of the cloud-based Internet. Moreover, it is introduced as an intermediate layer between mobile devices and the cloud in order to provide smooth, low-latency service delivery from the cloud to the mobile. In this paper, we combine a mobile ad hoc network with fog computing, and consider a new paradigm called a fog-based MANET, as shown in Figure 1.

Generally, MANET is called a mobile Ad hoc network, which is composed of many organizing mobile nodes or terminals via routing protocol. What’s more, the Ad hoc On-demand Distance Vector (AODV) protocol is just a typical routing protocol. Different from the clustered network, the mobile or fixed node can autonomously connect with the fog node, or construct a self-organizing, wireless, multi-hop, peer-to-peer, and dynamic mobile network, in which each node can act as a source node, a forwarding node, or a sink node. Each node is mobile, and can communicate with the single-hop node directly with wireless transmission technology. Then, its neighbor nodes decide how to transfer the data to the next hop, repeating this until reaching the fog node.

In the OpenFog Architecture Overview white paper for fog-computing, “Security implementations have many different descriptions and attributes such as privacy, anonymity, integrity, trust…”. To meet and achieve these requirements, the routing protocol in fog-based MANET aims at establishing high efficiency routing and transmitting information between nodes quickly. If any incorrect routing information is inserted into the network, or any routing information is tampered maliciously, the route will be misled, causing the whole network to become paralyzed. Hence, the security of the routing protocol is very important for the whole network. However, due to the characteristics of channel openness, dynamic topology, the lack of central authorization, distributed cooperation, and limited network capability, the traditional information security technologies that involve encryption [2], trust management [3], authentication [4], and secure network coding [5] are difficult to employ for solving the security issues of routing protocol directly and effectively.

As a traditional on-demand routing protocol in MANET, AODV (Ad hoc On-demand Distance Vector) routing protocol also faces the same security issue. This is because AODV builds on the fact that all nodes trust each other, and does not take any security into consideration [6]. Although some secure algorithms have been proposed to improve the AODV security, they pay little attention to the impact on transmission performance and energy efficiency. Furthermore, the anonymity is not considered for the source node in AODV. An attacker can explore and locate the source node by using traffic analysis. In this paper, a Source Anonymity-based Lightweight Secure AODV routing protocol (SAL-SAODV) is proposed to meet the above requirements, which involves defending against attacks and achieving energy efficiency in MANET. The rest of this paper is organized as follows: In Section 2, the related works are highlighted in AODV and its evolution. Some preliminary knowledge and a security analysis are represented in Section 3. The SAL-SAODV protocol is proposed, simulated, and analyzed in Section 4. Finally, some concluding remarks are given in Section 5.

## 2. Related Works

In this section, some of the research conducted so far related to AODV is introduced, which helps to build and support this paper. Currently, these researches are divided into two categories: one is focused on enhancing its security; the other is improving its performances (e.g., reliability, transmission performance under a dynamic topology).

### 2.1. Enhancing Security

Since the secure transmission does not take account of the design of the AODV protocol, the way in which we can solve the increasing number of security issues becomes very important. At present, many secure schemes and algorithms have been researched for AODV protocol. These schemes and algorithms mainly focus on detecting, defending, or mitigating against specific attacks, including the sinkhole attack, the blackhole attack, and the Sybil attack.

Ranjan summarized the security issues of MANET, especially under the blackhole attack [7]. The non-secure boundaries of the MANET made it vulnerable to various threats like information leakage by eavesdropping or the Denial of Services (DoS) attack. On the other hand, the lack of infrastructure and a central management system in MANET made it difficult to detect, defend, and mitigate the different security attacks.

Choudhurya et al. put forward a scheme to modify the AODV protocol in order to mitigate the blackhole attack in MANET [8]. The proposed scheme mainly created a Wait Time and Request Reply Tab table. Choudhary and Tharani demonstrated a timer-based detection approach for identifying a blackhole node [9], modified the code of the AODV protocol according to the blackhole attack procedure, and proposed a timer-based method to overhear the next node action in the network layer. The proposed approach could effectively remove a blackhole node from MANET. Bhandare et al. proposed a detection and defense scheme to eliminate the attacker, which carried out a blackhole attack [10]. The scheme could check the route reply against a fake reply based on “Normal V/S Abnormal (anomaly) activity”. Siddiqua et al. put forward a secure knowledge algorithm [11], which aimed to detect and prevent the blackhole attack in the promiscuous mode. Jain and Tokekar proposed a solution to defend against a blackhole attack based on the first *RREP* caching scheme in AODV protocol [12]. This solution could improve the performance in the routing protocol. Throug enhancing the protocol along with the context aware TOR (Trusted On-demand Routing) model, Hazra and Setua proposed a trust computation-based Sybil attack avoidance scheme in AODV [13]. The TOR model involved three major modules: the Node Manager, the Trust Module, and the Decision Manager. The Decision Manager secured the routing path on the basis of the trust value, which was computed in the Trust Module. The Node Manager reacted accordingly to AODV in response. Kasiran and Mohamad evaluated the throughput performance in AODV under a wormhole attack and Sybil attack [14], and gave the conclusion that the impact on throughput generated by the Sybil attack was greater than the impact of the wormhole attack. Patel et al. proposed an approach to detect the wormhole based on the Hash-based Compression Function (HCF) [15]. Actually, the approach used the secure hash function to compute a value of a hash field for the *RREQ* packet.

In addition, Ehsan and Khan implemented and analyzed attacks against MANET in NS-2 using AODV routing protocol. These attacks involved the blackhole attack, the sinkhole attack, the selfish node behavior, the *RREQ* flooding attack, the HELLO flood attack, and the selective forwarding attack [16]. They drew the conclusion that: if the malicious node was on the path from the source node to the destination node, the selective forwarding attack and the selfish node attack could cause a decline in the network performance. Furthermore, if the malicious node was connected to the source and destination, the sinkhole attack and the blackhole attack could severely affect the performance by transmitting false routing information and attracting all the traffic to themselves. Harwahyu et al. presented an analytical model [17] and estimated the performance of an AODV variant with a trust scheme. Chaubey et al. proposed a Trust-Based Secure on Demand Routing Protocol (TSDRP) [18], considered the Packet Delivery Fraction (PDF), the Average End-to-End Delay (AED), the Average Throughput (AT), and the Normalized Routing Load (NRL), and analyzed the impact of the pause time of TSDRP and AODV under the blackhole attack and the DoS attack in MANET.

From the above analysis, almost all of the secure algorithms and secure protocols in AODV have been proposed for efficiently detecting and defending/mitigating against the attacks in many different scenarios. However, it is regretful that these secure schemes could not defend against tramper attacks. Furthermore, these security technologies seemed to seldom consider the node’s energy consumption.

### 2.2. Improving Transmission Performance

As a distance vector routing protocol for MANET, AODV protocol permits intermediate nodes to reply, allowing the source node to quickly obtain routing in order to effectively reduce the number of broadcasts. Since nodes only store on-demand routing, this scheme can reduce the memory requirements and unnecessary duplications. However, because of periodically broadcast packets, a certain energy consumption and network bandwidth have to be considered. Due to the existence of stale routing, AODV protocol requires a relatively long latency to establish routes. Currently, the improvements of transmission performance in AODV mainly involve the following aspects: the energy efficiency, reducing the network congestion, improving the throughput, load balancing, and so on.

Su and Yang proposed Resilient AODV (RAODV) protocol [19]. In the route discovery phase, RAODV differed from AODV. RAODV protocol established as many routes as possible, whereas AODV only established one routing path from a source node to the destination node. Thus, when the primary route broke, the node could immediately adopt an alternative route without further route search. If there was no possible alternative route, the node would transmit the route break information backward to instruct the previous node on the reverse route to select an alternative one until an alternative route was found. The RAODV protocol could effectively reduce the number of route rediscovery procedures, the Packet Loss Rate (PLR), and the transmission latency, especially in sparse MANET. Liu et al. presented an optimized protocol, B-AODV [20], to solve the shortage of routing finding and routing repair in AODV protocol. There were two steps in B-AODV. Firstly, B*RREQ* (B-AODV *RREQ*) was used to replace of *RREQ* in order to reduce the time of route finding. Secondly, optimizing the two hops record in control messages and route table could improve the rate of routing repair and reduce the time required to find a route. Additionally, it improves the function of the Ad Hoc network. Hanji and Shettar introduced an Improved AODV (I-AODV) protocol [21]. In the I-AODV protocol, the location and energy level of the nodes were considered, and the route discovery area was restricted based on the source and destination location. The nodes lying in this region were taken into account as route nodes. One hop communication took place when the two nodes were located in the communicating range of each other. If the communicating nodes were far away each other, then according to the distance between the selected intermediate node and destination node, the energy for the intermediate nodes was greater than the threshold energy, and the intermediate nodes were re-selected. This scheme could increase the lifetime of the path. In addition, Apidet et al. evaluated the energy consumption and control response of the AODV protocol in WSANs for building-temperature control [22]. Carolina et al. evaluated and contracted the MAC/network/energy performance of MPH, AODV, DSR, and ZTR Routing Protocols in WSN [23].

For addressing the congestion issue in MANET, Bala and Krishna analyzed the performance of AODV and GPSR (Greedy Perimeter Stateless Routing) protocols in a VANET in multi-scenarios under different traffic conditions with respect to the Packet Delivery Ratio (PDR) and average End-to-End Delay (E2ED) [24]. They found that AODV performed better with respect to PDR and GPSR outperformed AODV with respect to E2ED. Moreover, the performance of both the routing protocols varied from one scenario to another and between traffic types. The performance of both AODV and GPSR was improved by using IEEE 802.11p instead of IEEE 802.11.

In improving the throughput, Hamidian proposed an extended AODV (AODV+) protocol [25], which allowed mobile nodes to communicate using the Internet. The AODV+ protocol had the ability to establish gateways, which support Vehicle-to-Infrastructure (V2I) communication. The proposed routing protocol can effectively improve the throughput. Because AODV+ protocol only considered how to connect the destination node in the Internet or infrastructure network, Wantoro and Mustika proposed a Modified AODV+ (M-AODV+) [26] to achieve reliable transmission from the mobile source to the destination in VANETs. This protocol involved three parts: communication channel selection, gateway discovery, and infrastructure to infrastructure communication routing.

For load balancing, Arya and Gandhi presented a node-disjoint AODV (NDj-AODV) [27] routing protocol that identified multiple node-disjoint routes to the destination. The proposed scheme could be used for load balancing, QoS- (Quality of Service) based routing, and more specifically, for performing energy aware routing. Regarding QoS, Sharma and Dimri proposed a routing protocol: AODV with QoS (QAODV) [28]. In QAODV, two additional fields were added to the message of AODV routing protocol in order to improve the performance in terms of PDR, PLR, and average E2ED.

Many valuable schemes in AODV are proposed to improve the transmission performance, including the energy efficiency, reducing the network congestion, improving the throughput, and load balancing. Just as mentioned above, there are many researches on enhancing the security and improving the performance in AODV. Unfortunately, joint works of the above two aspects are seldom researched. Moreover, we argue that the practical secure-routing-protocol is a tradeoff between security, energy consumption, and transmission performance. Therefore, we will propose a Source Anonymity-based Lightweight Secure AODV Protocol (SAL-SAODV) in the following sections in order to meet the security requirements of a fog-base MANET.

## 3. Preliminary Knowledge and Analysis

### 3.1. AODV

AODV (Ad hoc On Demand Distance Vector) was published as the experimental RFC of Ad hoc network routing protocol by IETF’s MANET working group in 2003. AODV is an on-demand algorithm. It draws on the broadcast routing discovery algorithm and routing maintenance scheme with DSR (Dynamic Source Routing). However, it’s not exactly a source routing protocol. It’s a large cost protocol because each data packet takes the source routing, so the AODV dynamically establishes a routing table entry by the intermediate nodes. It also employs DSDV (Destination Sequenced Distance Vector)’s hop-by-hop routing scheme and sequential numbering, and periodically keeps the routing information up-to-date in the route maintenance period. AODV efficiently avoids generating a loop by effectively using the sequence number of the destination node. Form the on-demand property, we can realise that: (1) nodes that are not on the route towards the destination node are neither involved in maintaining any routing information nor responsible for any periodical routing information exchange; (2) nodes do not need to discover and maintain routes to other nodes unless a communication needs to take place; (3) if the mobile nodes need a local connection, they obtain the routing information from the route discovery process. Generally, AODV protocol includes the following five aspects:

#### 3.1.1. Route Discovery

In the mobile ad hoc networks (MANET), when the source node must send a packet to the destination node, but there is no valid routing message about the destination node in its routing table, the source node starts a route discovery process to establish a route towards the destination node. Therefore, the source node sends its neighbors a route request message (*RREQ*). The neighbor nodes receive the request and forward it to their neighbors, so that the *RREQ*s are broadcasted using the flooding approach. Each node in MANET maintains two counters independently: the broadcast ID counter and the node sequence number counter. The *RREQ* message contains the broadcast ID and the source node address, and a *RREQ* message is thus uniquely identified.

A neighbor node receiving a *RREQ* message has three potential roles: (1) the neighbor node is the destination node. In this case, the *RREQ* message reaches the destination node and the neighbor node reacts with a route reply (*RREP*) message. Then, the *RREP* message is sent as a unicast towards the source node, using the path established by the *RREQ*. This process is similar to what happens with *RREQ*; (2) the neighbor node is not the destination node, but the neighbor node knows a route towards the destination. In this case, the neighbor node generates a *RREP* message and sends it to the source node, but the source node will deliver the *RREQ* message to the destination node if needed; (3) the source node is not the destination node and has no valid route to the destination node. The source node increases the hop count and forwards the message to its neighbors. It should be noted that a node may receive multiple *RREQ* messages with the same IP address and broadcast ID. Thus, when a node receives the *RREQ* message, it first checks whether the *RREQ* message has previously been received, and discards the *RREQ* message if it has. If a node is not the destination node, it has to take part of the *RREQ* message to set the reverse routing information, so that the other nodes can identify a route (the “reverse route”) toward the source node. If a node is not the destination node, it has to track part of the *RREQ* message to set the reverse routing information, so that the other nodes can identify a route (the “reverse route”) toward the source node.

#### 3.1.2. Route Maintenance

Each node maintains its own broadcast ID counter and sequence number counter. The sequence number counter is a 32-bit loop counter, and the nodes need to increment the sequence number count before an operation. The sequence number represents the new level of a node. AODV always favors newer information, and the nodes update their routing information, called an update function, if they receive a message with a sequence number higher than the last recorded one for that destination.

After the *RREQ* message is broadcasted from the source node, each intermediate node on the route towards the destination node automatically saves the reverse path back to the source node. The reverse path will be maintained for a certain time in the routing table, so that the *RREP* message can return to the source node along it.

#### 3.1.3. Setting the Immediate Nodes

When an immediate node M receives an *RREQ* message, it inserts the message into the route to the last node or updates the reverse route to the last node. Before M completes either of these actions, it checks whether it has ever received the message. If it has, M refuses to deal with the *RREQ* message and discards it directly. In this subroutine routing forward setting, we do not focus on this disorder case.

#### 3.1.4. Management of the Routing Table

All items in the routing table not only maintain the routing information about the source node, the destination node, the source node sequence number, and the destination node sequence number, but also hold some other routing information that has little relationship to the communication. This information is called the soft state of the routing. Each routing message maintains a timer, and all routing information expires or becomes invalid after a timeout and is removed from the routing table. The length of valid time determines the size of the network structure.

#### 3.1.5. Local Connectivity Management

Once a node receives a broadcast form a neighbor node, it calls upon the insert function or the update function to update the local routing table information, so that all of the latest information of the neighbor nodes will be included in the local routing table. If one node doesn’t broadcast any information to its neighbor nodes for a long time, it needs to broadcast a hello message to them. A hello message is a special *RREP* message, sent by the node initiatively, and contains the broadcast ID and sequence number of the node owners. The hello message only has a *TTL* (Time to Live) of 1, which ensures that it is not forwarded by its neighbor node. In other words, the hello message won’t be broadcast outside its neighbor range. Nodes that receive the hello message update the connection information with the node that sends it. Receiving the hello message from a new node or no longer receiving any message from the original node implies a change in the local network.

The main advantage of AODV is the ability to reduce the routing overhead. The others are the possibility to use an extension ring search to control the flooding of *RREQ* packets. In addition, the deployment of the destination sequence number allows nodes to pursue more updated routing. However, there are several issues worthy of attention in AODV protocol. Firstly, the two-way link and periodic link confirmation are required to detect link damage. Then, it needs to maintain the routing table. In particular, this flooding scheme might cause some security issues. Furthermore, the flooding attacks generated excessive traffic to the lead to DoS in MANET.

### 3.2. Secure AODV

The latest release of the SAODV (Secure AODV) RFC protocol was proposed by Manel Guerrero Zapata in September 2006 [29]. SAODV is a security extension of the AODV protocol, based on the public key cryptography of RSA. SAODV adds security fields to the extended AODV routing packets, and all of the end nodes and intermediate nodes need to authenticate the grouping, so that the SAODV can guarantee the security in the routing discovery process and the route maintaining process. SAODV uses a digital signature and hash chain scheme to protect the routing messages: the digital signature is used to ensure the integrity of non-mutable fields in the routing message, and the hash chain is used to keep the mutable field (such as the hop count) from being misrepresented.

#### 3.2.1. SAODV Hash Chain

The SAODV protocol specifies that the intermediate nodes need to complete hash operations in the hash fields in the message and increase the hop count before forwarding the routing message. In order to send an *RREQ* or *RREQ* message, each node needs to do the following two steps: (1) Randomly generate a hash seed, let the hash fields of the *RREQ* or *RREP* be the seed, and set the *Max_hop* of the route so that it is equal to *TTL*; (2) Select a hash function *h*(•) for the hash fields, and complete hash operations on the seed Max_hop times. After this, we get *h^Max_h^*^o*p*^, which is then written into the top of the hash fields (called *Top_hash*). Each node receiving the *RREQ* or the *RREP* also completes the hash operations *Max_hop* times on the hash fields by the *h*(•) in the message, to check if the result is equal to *Top_hash*; if equal, it means that the number of hops is not maliciously reduced. The type of *Hash*, *Top_hash*, *Hop*, and *Max_hop* are all defined as natural numbers.

#### 3.2.2. SAODV Digital Signature

SAODV routing messages (*RREQ*, *RREP*, *RERR*) are digitally signed, in order to guarantee their integrity and authenticity. Except for the hop count and the hash fields, other fields in the message should not be changed in the transfer process. The node generates a routing message and signs the changeless part with its private key. The node receives this message and verifies the signature with the sender’s public key. The type of key and sign are defined as natural numbers. The signature function and the validation function are expressed as a crypt and decrypt, respectively.

Gerri and Ghion proposed and implemented an SAODV scheme, called A-SAODV (Adaptive SAODV), based on an adaptive scheme, and optimized the performance. This protocol is intended for multi-threads, including two execution threads. One is dedicated to cryptographic operations, avoiding the blocking of other message processing. The other is used to complete all of the other functions, such as routing message processing, SAOVD routing table management, timeout management, SAOVD packet generation, and packet forwarding.

A-SAODV adopts the adaptive reply decision scheme to optimize the SAODV double signature feature. The intermediate node can determine whether to respond to the *RREQ* message according to its own load state, and it won’t reply to *RREP* when it is overburdened with dealing with the routing packet signature or authentication.

A-SAODV adopts the adaptive reply decision scheme to optimize the SAODV double signature feature. The intermediate node can determine whether to respond to the *RREQ* message according to its own load state, and it won’t reply to *RREP* when it is overburdened with dealing with the routing packet signature or authentication. A-SAODV also optimizes some other processes, except when adopting the adaptive reply decision scheme. For example, it uses a cache to store the new routing packets that have been signed and authenticated, adapts key ring management, and so on. All these optimizations have played a positive role in improving SAODV performance. However, there are some shortcomings in avoiding the routing group flooding, and reducing the node calculation and signature time.

### 3.3. Security Analysis

As there is no security scheme, AODV may be attacked by malicious nodes, compromised nodes, and selfish nodes. Malicious nodes refer to the nodes for which the attacker could not verify the legality of its own identity because of the lack of effective encryption information; compromised nodes refer to the nodes for which internal attackers could verify their identity as legal nodes and be trusted by other nodes, and this would launch an offensive within the network; selfish nodes refer to the nodes that tend to deny their own resources so that the other nodes cannot benefit from them, so as to save their own resources.

AODV does not specify any security measure, effectively assuming that there are no malicious nodes participating in the routing process, but many kinds of attacks are possible. AODV could be attacked by malicious nodes, compromised nodes, and selfish nodes. For our analysis, the AODV is vulnerable to the following categories of attacks:

Tampering attack: malicious nodes tamper with the routing information, causing the destination node to receive the wrong messages. For example, when a malicious node forwards *RREQ* messages, it falsifies the hop fields in the routing message, so that it has a larger opportunity to be chosen as the active route. Once the attack is successful, the malicious node can make any change on the packets it forwards to achieve a spiteful purpose.

Forgery attack: malicious nodes forge routing messages, causing the other nodes to receive the wrong messages. For example, a malicious node creates fake *RREP* messages to impersonate a destination node, obtaining the packets that should be sent to the real destination.

Routing replay attack: the malicious node sends an obsolete routing message, so that the nodes that receive the message update the related entries in their routing tables.

Black Hole Attack: a malicious node exploits the *RREQ* broadcast scheme and sends a fake *RREP* message, in which the hop count and destination node sequence number are forged, to claim that it is the destination node or has the best route towards the destination node. After receiving the *RREP* message, the source node establishes a route towards the malicious node.

Rushing Attack: as one type of DoS attack, the malicious node exploits the *RREQ* duplicate suppression scheme, sending the *RREQ* message to the destination node before the legitimate node through some special means (e.g., using the pre-established tunnels, increasing the transmit power, etc.). Then, the legitimate *RREQ* messages later received will be discarded. In this way, the source node will establish the route, including the malicious nodes, when the data passes through the malicious node, and it can be discarded or changed arbitrarily by the malicious node.

Tunneling attack: two malicious nodes collaborate to transmit the routing messages from another node through the tunnel between them, resulting in the illusion of communication links between the two nodes. Thus, the malicious nodes can easily insert the fork routing information into the other node’s routing message, and prevent the other nodes from forming the correct routing information.

The advantage of the SAODV protocol is that it can resist the external attacks well. The source authentication and integrity of the messages are provided by the public key algorithm, and each time the routing message arrives at a hop, it has to be carried through a public key calculation. As for the public key algorithm, the computational complexity is high and the energy consumption is also large, so the forwarding performance of the routing protocol is badly influenced. The double signature increases the packet length and the computational complexity of the nodes, and the effect is less important. In SAODV, the *RREQ* message generated by the source node is broadcasted using a flooding approach, and it will always propagate over a certain hop range unless it reaches a node that responds to it. In a complex or bad condition, the *RREQ* will spread in a larger scale, that is, there will be more *RREQ* messages spreading in the network. As the range of the *RREQ* message propagation increases, the energy consumption of the nodes increases accordingly. What’s more, the protocol is not perfect in detecting a tunneling attack and defending against a DoS attack.

## 4. Source Anonymity-Based Lightweight Secure AODV Protocol

In this section, we propose a source anonymity-based lightweight secure AODV Protocol, which involves three parts: a source anonymity algorithm, lightweight tamper-proof scheme, and random delayed transmitting scheme. Then, we simulate and analyze SAL-SOADV by using NS-2.

### 4.1. Source Anonymity-Based Lightweight Secure AODV Protocol

#### 4.1.1. Source Anonymity Algorithm

In SAL-SAODV, the pseudonym scheme is that in which the virtual prime IDs corresponding to the false IDs are generated to hide the nodes’ true ID with the nodes ID. In general, the node’s ID of MANET is 4 bytes, which ranges from 1 to 4,294,967,296 (0 is generally not used). The number of prime numbers within 1 billion is 50,847,534, which is able to meet the requirement of the pseudonym. The pseudo-code of the prime queue (false ID queue) generation algorithm is described in Algorithm 1. Although the number of loops is executed more frequently during the generation of primes, the work only needs to be performed once in this algorithm.

**Algorithm 1.** Prime queue generation algorithm.1: Set nodes’ maximum number (*N_MAX_*) in MANET, Initialize the prime queue *P_i_*= 0, Set *P*_1_ = 22: **for** (Set counter *A* = 1; *A <= N_MAX_*; *A* = *A* + 1)3:   **for** (Set counter *B* = 2; *B <=* [*A* square root] + 1; *A* = *A* + 1)4:      **if**
*A*/*B* = 0 **then**5:        Exit this loop6:      **else**7:        *P_A_*= *B*8:      **end if**9:   **end for**10: **end for**

**Figure 2:**
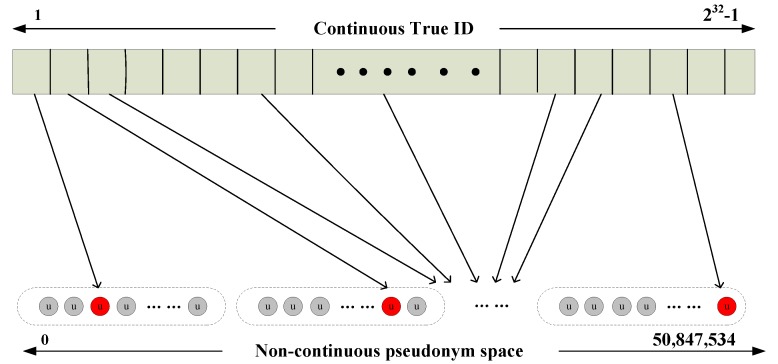
Non- consecutive pseudonym single mapping.

According to the false ID queue generated by Algorithm 1, the node can generate a false ID for itself. The false ID corresponds to the true ID in the false ID queue from the prime number sequence in the sequence of prime numbers, which is a single mapping in Figure 2. The *pseudonym* is generated by Formula (1).

(1) Pseudonmy=Rand(FalseID,FalseID+ℜ)

where, *Rand*(*n*, *n* + *m*) is a function, which can generate a random number between *n* and *n* + *m*. ℜ is a random number that is updated regularly. At this point, the false ID can be replaced with the generated pseudonym.

The process of index generation can be distributed in the initial stage of the network, by the signaling interaction between the sink node and each node, or by the operation of the node itself. In addition to the single mapping, the group mapping and block mapping and other tactics can be used to index and generate themselves false IDs, or a false ID set, and even the encryption can be implemented further. The specific mapping can be determined according to the actual requirements. In addition, the false ID can also be changed in the network operation process via the signaling interaction.

#### 4.1.2. Lightweight Tamper-Proof Scheme

The RSA digital signature in the SAODV protocol guarantees that the data is not tampered with, and the shortest key of the RSA digital signature is 1024-bit. Its computation and storage for the resource-constraint node are not a small overhead. In comparison, the deployment of Cyclic Redundancy Check (CRC) could also achieve a similar functionality—tamper-proof. What’s more, the polynomial of CRC-4 is only 5 bits. Meanwhile, the computational complexity of CRC is much lower than that of the RSA digital signature. So, we propose an Energy-efficient secure AODV protocol, which could effectively reduce the storage space and energy consumption of SAODV protocol, increasing the energy efficiency by using CRC instead of a RSA digital signature to test whether the data has been changed.

By attaching the gotten CRC check code at the back of the next original data and then making a transmission together, the receiver divides the data, and it represents data that has not been tampered with when there is no remainder.

CRC is an error detection code, which is usually used for detecting the unexpected data change in storage devices or the Internet. The data uses a CRC algorithm to obtain a check code, and this code is attached at the back of the transferred original data. Then, the receiver reuses the same CRC algorithm to check whether the data is being changed. Generally, the errors caused by environmental factors such as interference or harasses could use the CRC algorithm to effectively detect them in transmission. However, if the transmitting data and its CRC code is artificially manipulated at the same time by malicious nodes, it cannot not be detected whether the data has been tampered with the CRC algorithm.

In order to defend against the tamper attack, the polynomials are generated by a pseudorandom. Usually, the polynomial that is generated by the CRC algorithm is a fixed pattern, and the malicious nodes could be easy to tamper with the data and its CRC simultaneously, while if the pseudorandom polynomials are implemented, it could reduce the possibility of tampering with the data and its CRC. The transmitter and the receiver only need to negotiate and make the using sequence of the polynomial generated by pseudorandom in advance.

In addition, we could introduce the checksum (CHK) for verifying the CRC’s integration. Meanwhile, the checksum (1 byte) is encrypted by sharing the key, and thus the ability to defend against the tamper attack can be enhanced.

#### 4.1.3. Random Delayed Transmitting Scheme

In a sense, the point of using the RSA digital signature and CRC is to guarantee the data cannot be tampered with. Although the CRC is simple, and its security strength is not as high as RAS, when compared with the RAS digital signature (1024 bytes), CRC (4 bytes) has certain advantages in the computation and transmitted energy consumption, especially for resource-constrained nodes. Then, how to guarantee the integrity of CRC becomes a critical issue. In this sub-section, we give the random delayed transmitting scheme to solve the above problem, and use a checksum for the CRC code to guarantee its integrity to a certain extent.

In order to illustrate the proposed algorithm, the notations we have introduced and will introduce later are summarized in Table 1.

Briefly, the proposed scheme mainly involves two parts: generating the CRC and random delayed transmitting based on given random out-of-order space (*R_r_*). The random out-of-order space (*R_r_*) is generated by a Knuth-Durstenfeld Shuffle algorithm in fog node, and is described in Algorithm 2. Although the number of loops is executed more frequently during the generation of primes, the work only needs to be performed once in this algorithm.

**Algorithm 2.** To shuffle an array *a* of *n* elements (indices 0…*n*−1).1: **for**
*i* from *n*−1 down to 1 do2: *j* <- random integer with 0 <= *j* <= *i*3: exchange a[*j*] and a[*i*]

**Figure 3:**
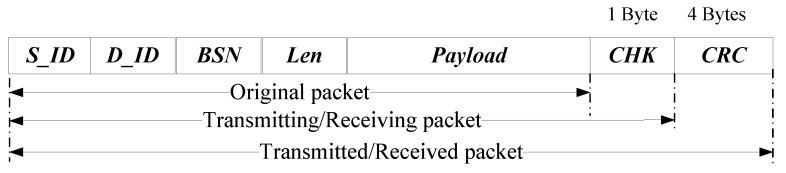
Structure of a Typical Data Packet in SAL-SAODV protocols.

In the SAL-SAODV, the structure of a typical data packet is shown in Figure 3. Meanwhile, we claim that intermediate nodes only constitute forward packets.

where, we define *R_r_* as a set, which includes the overall sequence randomly aligned 0 to 15. In theory, there are 16 possibilities for this permutation. Each aligned sequence has a strong randomness to randomize the order in which each packet matches the check information. By the following formula, a specific out-of-order sequence’s index is obtained.

**Figure 4:**
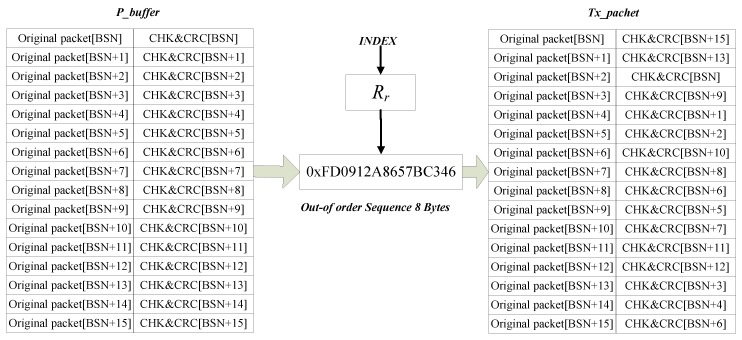
Random Delayed Transmitting Scheme.

*Index* = *P_s_* mod *P_d_* (2)

where, *P_s_* is the source node’s false ID and *P_d_* is the destination node’s false ID. These false IDs are generated by using Algorithm 1. A typical example is shown in Figure 4.

### 4.2. Simulation and Analysis

**Figure 5:**
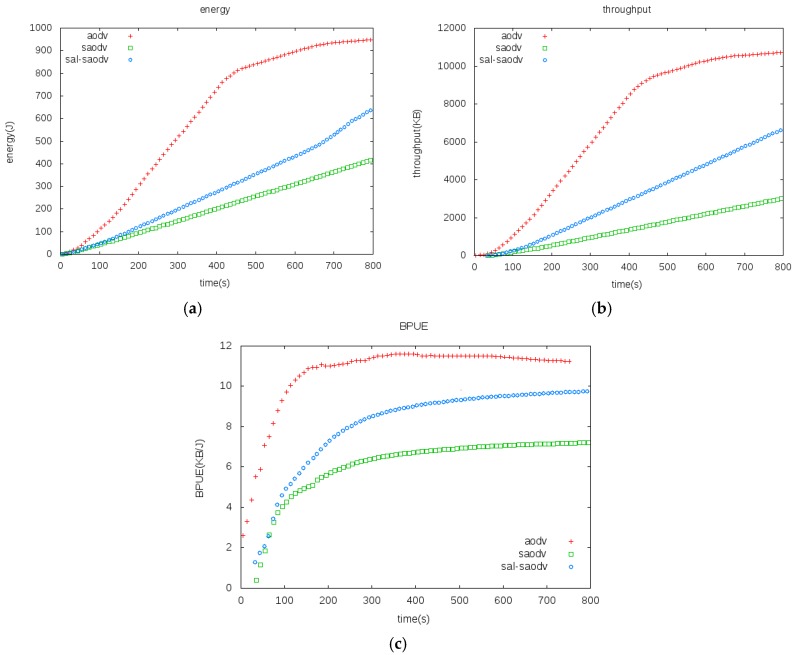
(**a**) Energy Consumption Comparisons, (**b**) throughput comparisons, (**c**) BPUE comparisons.

Simulation analysis is performed using a Network Simulator (NS-2), which is the most common tool for the simulation of network scenarios and topologies. We simulated AODV, SAODV, and SAL-SAODV protocol, and made a comparison in terms of energy consumption, throughput, and BPUE (Bits-Per Unit of Energy). BPUE is a multi-parameter joint evaluation metric based on the transmission distance and modulation level [30]. The simulation parameters are shown in Table 2:

The energy consumption of the three kinds of protocol is compared in Figure 5a. It can be seen from the diagram that the AODV protocol has the largest energy consumption, and the network energy consumption tends to be constant at about 700 s, which means that most of the nodes witness energy depletion and the network stops working; the SAODV protocol has the minimum energy consumption, and the SAODV and SAL-SAODV deal is still in a rising status in the 800 s, which means that the nodes have residual energy and the network can continue working.

The three protocols’ throughputs are compared in Figure 5b. It shows that the AODV’s throughput is bigger than SAODV and SAL-SAODV. It reaches its maximum at about 700 s, and its throughput is no longer high due to the fact that the network stops working, whereas SAODV’s throughput is the minimum.

Since AODV has no security schemes, its operating speed is the fastest. Meanwhile, its energy consumption and throughput exhibit the largest values. Instead, the SAODV protocol joins the security scheme and is more complicated than the SAL-SAODV protocol. Therefore, it exhibits the minimum energy consumption and throughput. The energy consumption and throughput values of the SAL-SAODV protocol are between the AODV and SAODV protocol. It can be concluded from the Figure 5a,b that the SAL-SAODV protocol can be used to reduce energy consumption by an average of 30% in terms of SAODV when they have the same throughput.

The BPUE of the three kinds of protocol is compared in Figure 5c. It can be seen from the diagram that the AODV protocol has the biggest BPUE, while SAODV displays the minimum; the BPUE of SAL-SAODV is between that of the AODV and SAODV protocol. It can be seen from Figure 5c that the BPUE metric is about 55% higher than that of the SAODV protocol when it remains steady. Figure 5a confirmed our viewpoint that the SAL-SAODV protocol improves the energy efficiency of SAODV.

## 5. Conclusions

Fog-base MANET is a special field of an Ad hoc network, with the internal architecture continuously extending in different directions based on instrumentation and a private network as basic components. Therefore, its own routing technology may be different from that of the general Ad hoc network. The fog-based MANET also produces higher security requirements, in addition to the technical features of highly heterogeneous, mobile characteristics. Therefore, fog-based MANET has a higher standard than a traditional Ad hoc network in terms of the security architecture, network security technology, secure routing protocol, privacy protection, security management, and guarantee measures. In this paper, it is proposed that the SAL-SAODV protocol is combined with fog-based MANET application scenarios based on SAODV protocol. By using a cyclic redundancy check instead of a digital signature to reduce the SAODV’s complexity and improve the energy efficiency of the protocol, the tamper-proof of packets is guaranteed by a random delayed transmitting mechanism. The simulation results show that the energy consumption is about 35% lower and BPUE is about 60% higher in the SAL-SAODV protocol than the SAODV protocol. At the same time, the schemes of increasing the energy efficiency and information tamper-proof could be used in the improvement of other AODV protocols. It is worth noting that the end-to-end latency will increase due to the random delayed transmitting mechanism. Hence, the SAL-SAODV protocol is suitable for the scenarios, which are delay-insensitive, but have a high security requirement.

## Figures and Tables

**Table 1:** Summary of notations.

Notation	Definition
*S_ID*	Source node ID
*D_ID*	Destination node ID
*BSN*	Block sequence number
*Len*	Length of original packet
*Payload*	Payload of packet
*CHK[BSN]*	Checknum for CRC
*CRC[BSN]*	Cyclic Redundancy Check for original packet
*P_buffer*	Transmitting/Receiving packets buffer
*C_buffer*	Transmitting/Receiving CRCs buffer
*Tx_packet*	Transmitted packet (involved: packet, *CHK* and *CRC*)
*Rx_packet*	Received packet (involved: packet, *CHK* and *CRC*)

**Table 2:** Simulation parameters.

Parameter	Value
Number of nodes	50
Initial energy of nodes (J)	2
Simulation area (m^2^)	1000 × 1000
Node movement speed (m/s)	0
Simulation time (s)	800
Transmission range(m)	200
Antenna Type	Omni antenna
Mobility Model	Random Way Point
